# Pericarditis Due to Campylobacter fetus subsp. fetus: A Case Report of an Uncommon Infection

**DOI:** 10.7759/cureus.48348

**Published:** 2023-11-06

**Authors:** Marília Andreia Fernandes, Francisco Gonçalves, Lino Gonçalves

**Affiliations:** 1 Department of Internal Medicine, Hospital Curry Cabral, Centro Hospitalar Universitário de Lisboa Central, Lisbon, PRT; 2 Department of Cardiology, Cardiac Intensive Care Unit, Coimbra University Hospital Centre, Coimbra Institute for Clinical and Biomedical Research (ICBR) Faculty of Medicine, University of Coimbra, Coimbra, PRT

**Keywords:** pericarditis, pericardial effusion, campylobacter fetus, campylobacter fetus subsp fetus, immunosuppression

## Abstract

Pericarditis is a common condition with numerous aetiologies. Bacteria other than the *Mycobacterium tuberculosis *complex are an exceptional cause. We present a case of subacute pericarditis highly probable due to *Campylobacter fetus *subsp. *fetus *in an immunosuppressed patient undergoing biologic therapy in relation to systemic lupus erythematosus (SLE). On admission, the patient presented with chest pain, dyspnea, and diaphoresis and has lately developed fever and a large pericardial effusion (PE) with a concomitant increase in the inflammatory parameters. The clinical presentation, along with the exclusion of a flare of the autoimmune disease and the isolation of *Campylobacter fetus *subsp.* fetus *on blood samples permitted the diagnosis. After therapy with antibiotics and colchicine, the patient showed full recovery.

## Introduction

Pericarditis is the most common disease affecting the pericardium. Infections, autoimmune diseases, malignancy, metabolic, iatrogenic, and drug-related conditions may cause inflammation of the pericardial sac, which has an unknown aetiology in most cases. In developed countries, bacteria are hardly ever implicated as a cause, except in those where tuberculosis has a high prevalence [1]. *Campylobacter* infections typically involve the gastrointestinal tract. However, invasive disease may sporadically occur, with *Campylobacter fetus* being the most common implied species (up to 53% of infections). Immunosuppression and regular contact with natural reservoirs are risk factors for bacteremia due to *Campylobacter fetus* [2]. We report a case of pericarditis due to *Campylobacter fetus* subsp. *fetus*, a rare infection with a dozen cases previously described in the literature [3].

## Case presentation

The patient was a 50-year-old female living in a rural area who was a smoker and had arterial hypertension, dyslipidemia, grade 1 obesity, peripheral arterial disease, and systemic lupus erythematosus (SLE) with nephritis, which was diagnosed 14 years ago. In the context of this diagnosis, she was medicated with prednisone 5 mg daily, hydroxychloroquine 400 mg daily, and belimumab 10 mg/kg monthly. She had no known allergies or other relevant medical history, including recent history of infection.

**Figure 1:**
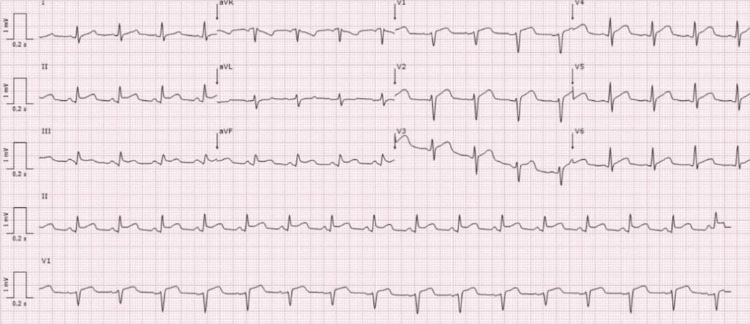
The 12-lead electrocardiogram performed at admission showing a concave ST-segment elevation in leads II, III, aVF, and V2-V4

The patient presented to the emergency department due to oppressive chest pain with several days of evolution and worsening in the previous four hours associated with dyspnea. At admission, her blood pressure was 85/60 mmHg, heart rate 100 beats per minute, temperature 35.4ºC, and oxygen saturation 96% on room air. She was diaphoretic and tachypneic, with no other relevant findings on physical examination. The 12-lead electrocardiogram (ECG) showed a diffuse concave ST-segment elevation (Figure 1). The blood analysis denoted an elevation of leucocytes (10.95 x 10^9^/L, reference range 4.0-10.0) and C-reactive protein (CRP, 113.1 mg/L, <5.0) without alteration of haemoglobin, platelets, troponin I, creatine kinase, urea, and creatinine serum levels. The chest radiograph was also normal. Even so, suspecting an ST-elevation acute coronary syndrome (ACS) in a centre where percutaneous coronary intervention (PCI) is not available and predicting an absolute time from diagnosis to PCI-mediated reperfusion greater than 120 minutes, the patient underwent systemic fibrinolysis with tenecteplase 10,000 units. Afterward, she was transported by helicopter to the closest centre able to perform an urgent PCI.

**Figure 2:**
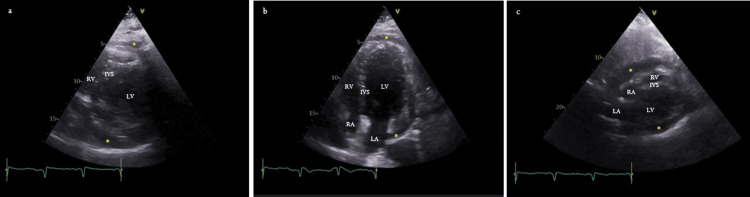
Transthoracic echocardiogram demonstrates a moderate circumferential pericardial effusion (asterisks), measuring 15 mm (a) Parasternal short axis. (b) Apical four-chamber view. (c) Subcostal view. LV: left ventricle, RV: right ventricle, LA: left atrium, RA: right atrium, IVS: interventricular septum

Besides minor irregularities in the left anterior descending and circumflex arteries, the coronary angiogram revealed an ostial stenosis of 91-99% in the right coronary artery (dominant). A drug-coated stent was placed in the stenotic lesion with a good angiographic result. Nonetheless, during the first 48 hours of the in-patient period, the patient was still complaining about strong thoracalgia, especially when lying down, and high fever. By that time, inflammatory parameters had increased (leucocytes 12.4 x 10^9^/L, CRP 241.4 mg/L, and erythrocyte sedimentation rate (ESR) 99 mm/h, 1-20), keeping myocardial injury and renal function markers within normal range. The patient had performed the first transthoracic echocardiogram, which revealed a mild pericardial effusion (PE) with no wall motion abnormalities. These findings corroborated the results of recently made thoracic computed tomography angiography, which incidentally showed a spontaneously dense, non-loculated fluid surrounding the left ventricle. After discussion with her rheumatologist, the study was also completed by dosing complement, anti-double-stranded DNA (anti-dsDNA), and anti-ribonucleoprotein (anti-RNP), all within the normal range. Thus, the patient started colchicine 0.5 mg twice daily and increased aspirin to 750 mg every eight hours, keeping the usual corticosteroid dose, considering disease flare unlikely according to the SLE Disease Activity Index 2000 (score increasing of three points compared with the previous assessment and counting with fever, whose infectious aetiology hasn’t been ruled out yet). One week later, the echocardiogram pointed out an increased PE with 15 mm, circumferential, and no haemodynamic compromise (Figure 2). Afterward, serial echocardiograms were performed until documentation of improvement of PE (maximum 23 mm with no signs of cardiac tamponade; at discharge, just a small lamina, mainly posterior). In the meantime, *Campylobacter fetus* subsp. *fetus* was isolated in blood cultures, and the patient started gentamicin 5 mg/kg/day and ceftriaxone 2 g/day. After 10 and 15 days, in this order, she stopped the aforementioned antibiotics, given the good clinical and laboratory evolution with demonstration of clear blood cultures. The patient was discharged after 26 days of hospitalisation with the indication to maintain colchicine and avoid vigorous physical activity for three months. Three months after leaving the hospital, the patient was asymptomatic, with echocardiographic evidence of total resolution of PE.

## Discussion

Acute pericarditis (AP) usually manifests as sharp and pleuritic chest pain, which is relieved by sitting upright and leaning forward, and a widespread ST elevation (hallmark sign) or PR depression on ECG. A pericardial rub may be audible, and a new PE may occur in up to one-third and 60% of patients, respectively. Elevation of inflammation markers and imaging evidence of pericardial inflammation support the diagnosis of AP [1]. AP imposes a differential diagnosis with ACS [1], which may not be straightforward in patients who have multiple cardiovascular risk factors and present the so-called angina equivalents [4], namely dyspnea and diaphoresis, as in this case. Indeed, some hospitals do not have the availability to perform urgent PCI (like the one where the patient had been initially admitted), which diminishes the time to make a decision and meet the recommended time to implement a reperfusion strategy.

Since the diagnosis of AP is established, the presence of high-risk features should be looked for, as the presence of any determines the need for hospitalisation. An aetiologic study is mandatory in those cases [1]. Viral infections are the most common cause of AP [1], accounting for around 90% of cases, together with those that have an unexplained aetiology [5]. Systemic autoimmune diseases could be responsible for 15% of acute or recurrent pericarditis [1]. In fact, serositis is a usual manifestation of the extra-renal flare of SLE [6], and pericarditis could reach up to 54% of the patients [7]. Among others, nephrotic syndrome with renal insufficiency, interstitial lung disease, pulmonary hypertension, and cardiomyopathy have been associated with pericarditis in patients with SLE, but none of these features were evident in our case. Furthermore, excluding fever and high ESR (which in fact could also be attributable to other causes), the absence of clinical and serological signs of disease flare (namely, haemolytic anaemia, thrombocytopenia, elevation of anti-dsDNA and/or anti-RNP, and low complement) [6,7] makes lupus PE less probable. On the other hand, immunosuppressive treatment related to this disease may make the patient prone to opportunistic microorganisms [1], like *Campylobacter* *fetus* subsp. *fetus* [2,5] lately been identified. Of note, despite the fact that bacterial AP is fairly uncommon, tuberculosis is the most frequent form of pericardial disease in developing countries and the most prevalent cause of AP in developed countries with a high prevalence of this infection [1], such as Portugal [8]. Actually, the suspicion of purulent pericarditis should promptly motivate the performance of a pericardiocentesis, which permits the drainage essential for the treatment, and the biochemical and cultural analysis of pericardial fluid samples collected. Nonetheless, this procedure wasn’t performed, considering the lower estimated content of pericardial fluid in a patient under dual antiplatelet therapy. Indeed, non-steroidal anti-inflammatory drugs (NSAIDs) are used as first-line treatment for AP, with ibuprofen being the drug of choice, unless ischemic heart disease or other indications for antiplatelet treatment are present when aspirin ranks first. This class of drugs should be kept accordingly until evidence of inactive disease, which is determined by full symptom remission and CRP normalisation. In turn, colchicine, which is an adjuvant drug that avoids recurrence, should be maintained for three months. Corticosteroids should only be used as an alternative option in patients with contraindications or failure of NSAIDs or when a systemic disease is present, as they are linked to the chronic evolution and recurrence of the disease. Moreover, specific therapy for the underlying cause is indicated. Regarding non-pharmacological measures, physical activity should be restricted to less than usual until disease remission in non-athletes [1].

AP does usually have a benign course. However, there are some factors that predict an ominous prognosis. Subacute onset, high fever, large PE, failure to respond within seven days to NSAIDs, and immunosuppression, all present in the case reported, are related to a poorer prognosis. Additionally, bacterial pericarditis has an increased risk of cardiac tamponade, recurrence, and constriction, which confer greater mortality and morbidity [1].

## Conclusions

Pericarditis is a clinical diagnosis supported by laboratory and imaging data. Personal history may lead to the most likely aetiology, which is useful to search for as it influences accurate management. Immunosuppressive conditions should lower the threshold of suspicion for less common infections, especially when they impose a non-neglected risk of mortality and/or morbidity.
